# CARD14 Expression in Dermal Endothelial Cells in Psoriasis

**DOI:** 10.1371/journal.pone.0111255

**Published:** 2014-11-04

**Authors:** Jamie L. Harden, Steven M. Lewis, Katherine C. Pierson, Mayte Suárez-Fariñas, Tim Lentini, Francesca S. Ortenzio, Lisa C. Zaba, Raphaela Goldbach-Mansky, Anne M. Bowcock, Michelle A. Lowes

**Affiliations:** 1 Laboratory for Investigative Dermatology, The Rockefeller University, New York, New York, United States of America; 2 Center for Clinical and Translational Science, The Rockefeller University, New York, New York, United States of America; 3 Translational Autoinflammatory Disease Section NIAMS/NIH, Bethesda, MD, United States of America; 4 Genomic Medicine, Imperial College London, London, England; University of Tennessee, United States of America

## Abstract

Mutations in the *caspase recruitment domain, family member 14* (*CARD14*) gene have recently been described in psoriasis patients, and explain the *psoriasis susceptibility locus 2* (*PSORS2*). CARD14 is a scaffolding protein that regulates NF-κB activation, and psoriasis-associated *CARD14* mutations lead to enhanced NF-κB signaling. CARD14 is expressed mainly in epidermal keratinocytes, but also in unidentified dermal cells. In this manuscript, the identity of the dermal cell types expressing CARD14, as well the potential functional consequence of overactive CARD14 in these dermal cell types, was determined. Using two-color immunofluorescence, dermal CARD14 did not co-localize with T-cells, dendritic cells, or macrophages. However, dermal CARD14 did highly co-localize with CD31^+^ endothelial cells (ECs). CARD14 was also expressed non-dermal endothelial cells, such as aortic endothelial cells, which may indicate a role of CARD14^+^ECs in the systemic inflammation and cardiovascular comorbidities associated with psoriasis. Additionally, phosphorylated NF-κB was found in psoriatic CARD14^+^ CD31^+^ ECs, demonstrating this pathway is active in dermal ECs in psoriasis. Transfection of dermal ECs with psoriasis-associated *CARD14* mutations resulted in increased expression of several chemokines, including CXCL10, IL-8, and CCL2. These results provide preliminary evidence that CARD14 expression in ECs may contribute to psoriasis through increased expression of chemokines and facilitating recruitment of immune cells into skin.

## Introduction

Psoriasis vulgaris is a chronic inflammatory skin disease, characterized by activated hyperproliferative keratinocytes and infiltrating immune cells, specifically Th17 and Th1 cells, and inflammatory dendritic cells [1]. Although the precise etiology of psoriasis is not completely understood, several genetic factors predispose individuals to this chronic skin disease. These chromosomal regions are identified as *psoriasis susceptibility loci* (*PSORS1-PSORS16*). However, the exact gene (or genes) responsible for the susceptibility within each *PSORS* region have not yet been identified.

Recently, Jordan *et al* described mutations in the *caspase recruitment domain family, member 14 (CARD14/CARMA2*) gene that are responsible for *PSORS2* [2]. Mutations in *CARD14* were described in two families (one of European descent, and a Taiwanese family), as well as a *de no*vo mutation in a Haitian child with sporadic, early-onset, generalized pustular psoriasis [2]. Additionally, a SNP in *CARD14* achieved genome-wide significance in a recent meta-GWAS study containing 10,588 cases of psoriasis and 22,806 controls [3]. CARD14 mRNA was also differentially upregulated in lesional psoriasis in an RNAseq study [4]. Together, these observations suggest CARD14 may play a pathogenic role not only in individuals with psoriasis associated *CARD14* mutations, but also in general psoriasis pathogenesis.

CARD14 is a scaffolding protein involved in activation of nuclear factor-kappa B (NF-κB). In steady state conditions, NF-κB is located within the cytoplasm, bound by the inhibitor IκB [5]. Upon activation of NF-κB signaling, IκB is phosphorylated by IκB kinase (IKK), targeting IκB for degradation, allowing release and activation of NF-κB [5], [6]. Related CARD family members interact with molecules involved in NF-κB signaling, such as Bcl10, MALT1, and TRAF2, which aid in recruitment and activation of the IKK complex [5], [7], [8], and CARD14 might be operating in the same way. The familial psoriasis-associated mutations in *CARD14* result in altered splicing and inclusion of an additional 22 amino acids within the CARD14 protein (G117S) [2], [9]. The *de novo* mutation is a missense point mutation (E138A) [2]. Both of these mutations result in production of a gain-of-function CARD protein and subsequent overactive NF-κB signaling, with the E138A mutation resulting in the most dramatic increase in NF-κB activity [2], [9]. However, the molecular details of how these CARD14 mutations increase NF-κB signaling and lead to inflammation have not yet been elucidated.

CARD14 was originally described as most abundant in placental and mucosal tissue [8]. Recently, our studies have demonstrated that CARD14 is expressed in epidermal keratinocytes [2], [10]. Transfection of keratinocytes with the psoriasis-associated mutations in *CARD14* resulted in increased NF-κB activity, as well as transcription of pro-inflammatory genes, such as CCL20 and IL-8 [2], [9]. However, previously published immunohistochemistry also revealed a dermal population of CARD14-expressing cells, but the identity of this population has not yet been investigated [2].

In this study, we found that dermal CARD14 localized to the CD31^+^endothelial cells (ECs). Both dermal blood and lymphatic ECs were found to express CARD14, and dermal ECs co-localized with phosphorylated NF-κB (pNF-κB), indicating activity of this signaling pathway in ECs within psoriatic skin. Transfection of ECs with psoriasis-associated CARD14 mutations resulted in increased expression of several chemokines, including IL-8, CCL2, and CXCL10. Culture of dermal ECs with psoriatic cytokines (such as IFNγ, IL-17, and TNFα) also induced expression of many of the same chemokines found upregulated after transfection of ECs with psoriasis-associated CARD14 mutations. Additionally, several of these chemokines were indeed increased in lesional psoriatic skin, and co-localized with dermal ECs *in situ*. Similar findings were observed in both classical psoriasis patients and a patient from a PSORS2-linked family (GEN001), who harbored a confirmed gain-of-function *CARD14* mutation (G117S). Therefore, CARD14 may play an important role in psoriasis pathogenesis partially through pro-inflammatory activities within dermal endothelial cells.

## Results

### Dermal CARD14 is expressed in endothelial cells

**Figure 1:**
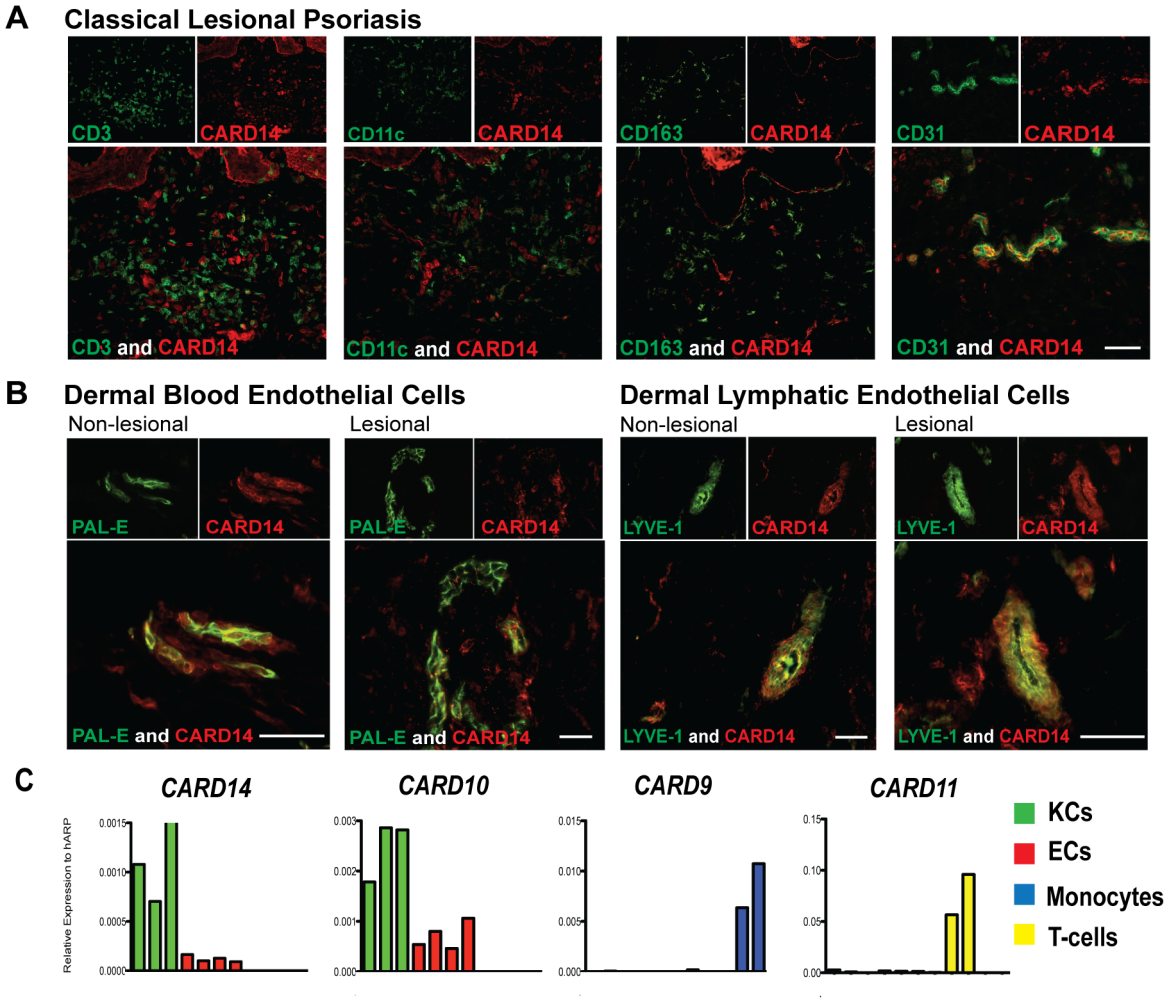
CARD14 was expressed in dermal endothelial cells. Two color immunofluorescence on frozen sections of psoriatic skin for CARD14 (red) versus (a) CD3^+^T-cells, CD11c^+^ dendritic cells, CD163^+^ macrophages, and CD31+ endothelial cells (green); as well as versus (b) PAL-E+ blood endothelial cells and LYVE-1 lymphatic endothelial cells (green). Representative images; bar = 10 µm. (c) Quantitative RT-PCR analysis of various CARDs in keratinocytes (green), endothelial cells (red), T-cells (blue), and monocytes (yellow).

Previous studies have demonstrated that CARD14 is expressed in epidermal keratinocytes [2]. However, the presence of a population of dermal CARD14^+^ cells was noted in these studies ([2]; Figure S1 in File S1). To determine which cell types within normal, non-lesional (NL), and lesional (LS) psoriatic skin expressed CARD14, double immunofluorescence studies were performed. Due to the critical role of activated immune cells in psoriasis, we first determined if CARD14 co-localized with markers of various immune cell populations. Dermal CARD14 did not co-localize with CD3 (T-cells), CD11c (dendritic cells), and CD163 (macrophages) in all samples (Figure 1a and Figure S2 in File S1), indicating that these immune cells do not express CARD14.

Noting the pattern of CARD14 expression, we considered that CARD14 protein might identify a vascular cell population. Indeed, CARD14^+^ cells were highly co-localized with the general EC marker, CD31 (Figure 1a and Figure S2 in File S1), most notably in lesional skin, where there are abundant blood vessels. ECs (CD31^+^ cells) can be further subdivided into two types: human dermal blood endothelial cells (HDBECs) and human dermal lymphatic endothelial cells (HDLECs), defined by expression of PAL-E and LYVE-1, respectively. CARD14 co-localized with both LYVE-1^+^ cells and PAL-E^+^ cells (Figure 1b), indicating expression within both types of ECs. CARD14 also co-localized with vimentin, a marker of mesenchymal cells (Figure S3a in File S1). Triple immunofluorescence studies showed that vimentin^+^CARD14^+^ cells were also CD31^+^ (Figure S3b in File S1). These studies demonstrated that ECs were indeed the dermal cell type expressing CARD14.

### CARD mRNA in Keratinocytes, Endothelial Cells, T-cells, and Monocytes

To validate these immunofluorescence findings, quantitative RT-PCR analysis was performed for expression of CARDs in various cell populations (Figure 1c). CARD14 was detected at highest amounts in keratinocytes (KCs), however, both HDBECs and HDLECs also expressed CARD14 mRNA. CARD14 mRNA was not detected in T-cells or monocytes. CARD10 is expressed in non-hematopoietic cells [5], and therefore served as a positive control in keratinocytes and ECs. CARD9 and CARD11, which are highly expressed by monocytes and T-cells, respectively [5], were minimally detected in KCs and ECs. These findings confirm that CARD14 is expressed in both KCs and ECs.

### CARD14 in aortic tissues

To determine if endothelial cells outside of the skin might express CARD14, RNA from human aortic endothelial cells (HAECs) was assessed for expression of CARDs. Similar to the HDBECs and HDLECs, HAECs expressed CARD10 and CARD14, albeit with lower levels of CARD14 mRNA expression compared to the dermal EC subsets, and minimal to no CARD9 or CARD11 (data not shown). In macroscopically identified NL and LS atherosclerotic aortas, CARD14 protein was significantly detected, with the clearest staining in the EC layer (Figure S4a in File S1). Immunofluorescence studies confirmed that CARD14 protein co-localized with the CD31^+^ aortic ECs (Figure S4b in File S1). This finding is particularly intriguing due to the cardiovascular comorbidities associated with psoriasis [11].

### Dermal Endothelial Cells Contain Active *NF-κB* Signaling in psoriatic skin

**Figure 2:**
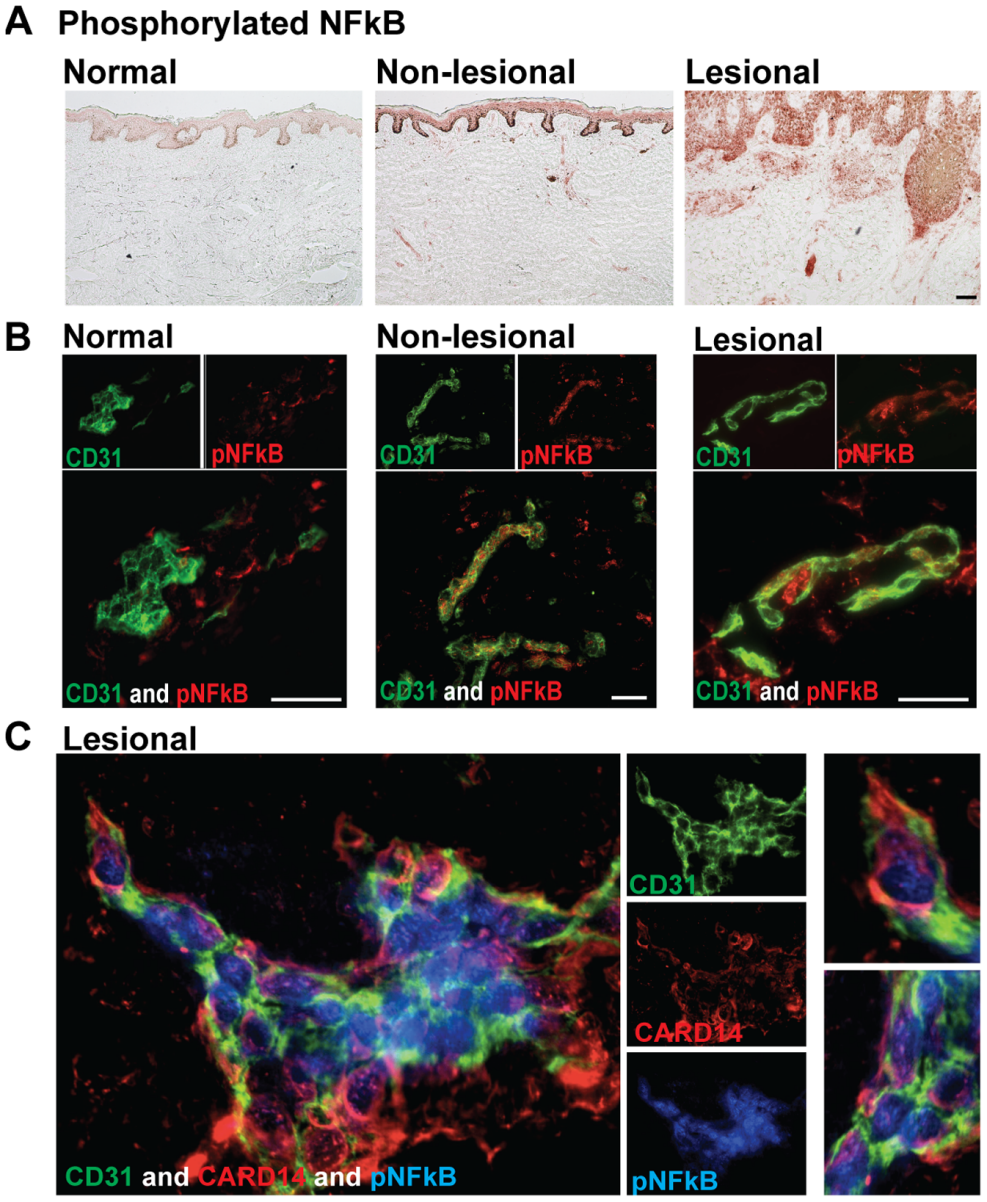
Phosphorylated NF-κB (pNF-κB) was upregulated in psoriatic skin and dermal pNF-κB co-localized with CARD14^+^ ECs. (a) Immunohistochemistry of pNF-κB in normal, non-lesional, and lesional frozen skin sections. (b) Two-color immunofluorescence of CD31 (green) and pNF-κB (red) in normal, non-lesional, and lesional skin. Representative images; bar = 10 µm. (c) Triple-color immunofluorescence staining of CD31 (green), CARD14 (red), and pNF-κB (blue) in lesional skin. Representative images at 63X magnification; enlarged images are shown to the left.

Active NF-κB signaling has been shown previously in psoriatic lesional skin by staining for pNF-κB [12], [13]. These studies focused primarily on pNF-κB in keratinocytes, showing significant staining of the epidermis in LS skin. However, dermal staining for pNF-κB was also observed. To confirm these findings, we performed immunohistochemistry for pNF-κB on normal, NL, and LS skin (Figure 2a). As previously published, normal skin had minimal pNF-κB, NL skin contained moderate amounts of pNF-κB, and LS skin contained the maximal amount of activated NF-κB. Dermal staining was most pronounced in LS skin. To determine if any of the dermal pNF-κB was within endothelial cells, we performed double immunofluorescence studies. Minimal expression of dermal pNF-κB and therefore minimal co-localization with CD31^+^ ECs was found in normal skin (Figure 2a–b). However, pNF-κB co-localized with CD31^+^ ECs in the dermis of NL and LS skin, showing that ECs exhibit an activated phenotype in psoriasis (Figure 2b). Triple immunofluorescence studies confirmed that the CARD14^+^ CD31^+^ dermal ECs in LS skin contained pNF-κB (Figure 2c). Expression of pNFkB in CD31^+^ dermal ECs was also confirmed using confocal microscopy (data not shown). In summary, these studies show that CD31^+^ECs contain the NF-κB scaffolding molecule, CARD14, and have phosphorylated NF-κB in LS and NL skin.

### Individuals with CARD14 mutations have similar skin transcriptomic and cellular profiles compared with classical psoriasis

In order to further evaluate the relationship between the patients with the *CARD14* mutations and classic psoriasis, their transcriptomes were compared. LS and NL skin from an adult patient with a familial G117S mutation in *CARD14* leading to a 22 amino acid insertion (“GEN001”) was obtained [2]. LS skin from a pediatric patient with the *de novo* E138A missense point mutation in *CARD14* (“NIH”) as well as a skin biopsy three months after successful treatment with anti-IL-12/23p40 treatment (ustekinumab, Stelara, Janssen) (post) were also collected. The principal components analysis (PCA) showed that the transcriptome of LS biopsies from patients with *CARD14* mutations clustered close to those of LS classic psoriasis, and the NL and post-treatment biopsies of these two patients clustered close to normal and NL skin of classic psoriasis (Figure S5a in File S1). Differentially expressed genes (DEGs) for these patients were defined as LS skin compared to the NL (for GEN001) or post (for NIH) skin. Gene Set Enrichment Analysis (GSEA; [14]) showed that published upregulated and downregulated psoriasis transcriptomes were significantly enriched in the transcriptomes of these two patients with *CARD14* mutations (Connectivity Scores of 0.69–0.78) (Figure S5b in File S1). The transcriptome of these two patients with *CARD14* mutations was mined using Ingenuity Pathway Analysis, to identify biologically relevant pathways enriched in these transcriptomes, and compared with the psoriasis meta-analysis derived (MAD3) transcriptome (Top pathways shown in Figure S6a in File S1) [15]. Many of these pathways have been previously shown to be upregulated in psoriasis, such as *Atherosclerosis Signaling*, and *Role of IL-17A in Psoriasis*. An estimation of expression of many other cytokine gene sets found in psoriasis shows that they were similar in these two patients (Figure S6b in File S1).

**Figure 3:**
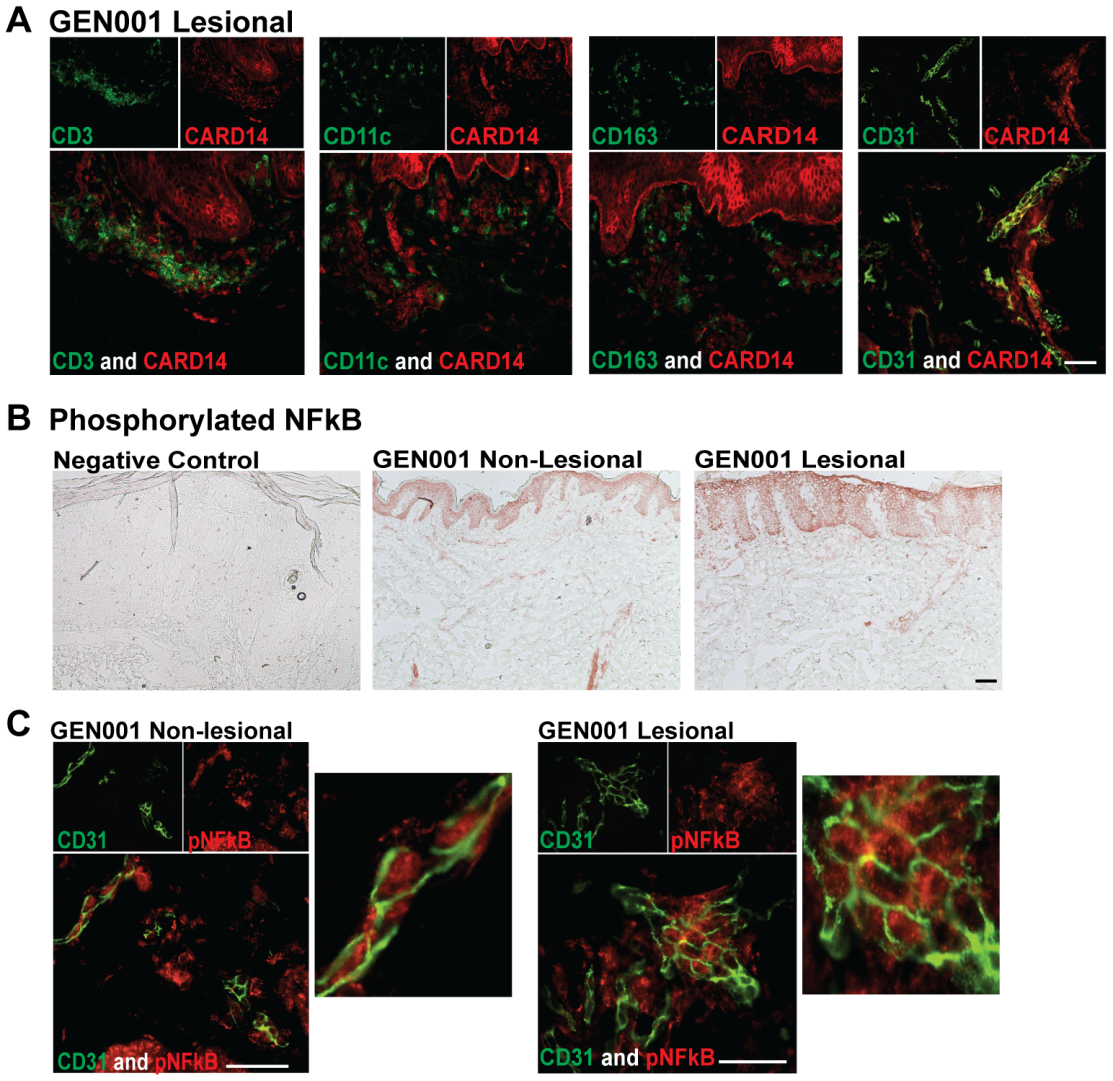
Patient with a confirmed *CARD14* mutation expressed CARD14 and activated NF-κB in dermal endothelial cells. (a) Two color immunofluorescence of lesional skin for patient GEN001 (harbors an activating *CARD14* mutation) for CARD14 (red) versus CD3^+^ T-cells, CD11c^+^ dendritic cells, CD163^+^ macrophages, and CD31^+^ endothelial cells (green). (b) Immunohistochemistry of phosphorylated NF-κB (pNF-κB) in non-lesional (middle) and lesional (right) frozen skin sections from patient GEN001 [2]. Negative-control immunohistochemistry staining is shown in lesional psoriatic skin (left). (c) Two-color immunofluorescence staining of CD31 (green) and pNF-κB (red) in non-lesional (left) and lesional (right) frozen skin sections from patient GEN001. Enlarged images are shown to the left. Bar = 10 µm.

Histologically, NL and LS skin biopsies from GEN001 were indistinguishable from classic psoriasis [2], and contained similar numbers of CD3^+^ CD11c^+^ and CD163^+^ cells (data not shown). Two-color immunofluorescence studies were also similar to the findings in classical psoriasis. GEN001 LS skin contained CARD14^+^ dermal cells that co-localized with CD31^+^ECs, but not with CD3 (T-cells), CD11c (dendritic cells), or CD163 (macrophages) (Figure 3a). GEN001 NL and LS skin also contained active pNF-κB signaling (Figure 3b). As was shown in classical psoriasis, dermal pNF-κB also co-localized with endothelial cells in GEN001 NL and LS skin (Figure 3c). In conclusion, these findings suggest that individuals with CARD14 mutations exhibit a typical psoriasis phenotype, as substantiated by transcriptome profiling, immunostaining, and expression of CARD14^+^ and pNF-κB^+^ ECs.

### Overactive CARD14 increases chemokine expression in HDBECs

**Figure 4:**
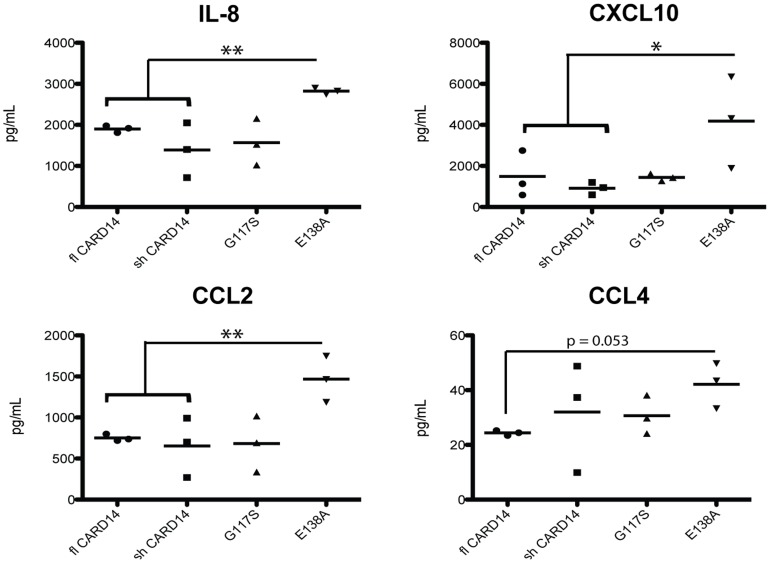
Transfection of psoriasis-associated *CARD14* mutations into dermal endothelial cells resulted in increased expression and of several chemokines. Protein expression of various chemokines in cell culture media of endothelial cells transfected with wild-type (fl and sh) or psoriasis-associated *CARD14* mutation constructs (G117S and E138A) and stimulated overnight with TNFα (25 ng/mL). N = 3 per group. * P<0.05, ** P<0.01.

ECs produce chemokines that recruit immune cells to sites of inflammation. To determine the functional effect of CARD14 activity in dermal ECs, psoriasis-associated (overactive) CARD14, as well as wild-type (non-overactive) CARD14 plasmid constructs, were transfected into HDBECs. The overactive CARD14 constructs contain mutations that were described previously in patients, specifically the G117S mutation that results in a 22 amino-acid insertion within the CARD14 protein, and the highly overactive E138A mutation [2]. Supernatants from wild-type or mutant CARD14 transfected HDBECs were assayed for chemokine production using a multiplex cytokine array. Mutant E138A CARD14 transfected HDBECs secreted significantly more IL-8, CXCL10, and CCL2 protein compared to wild-type CARD14 HDBECs (Fig 4). CCL4 protein expression was also almost significantly increased in the E138A mutation. No differences were found in production of Eotaxin, CCL17, and MCP-4 (data not shown). Additionally, expression of *CCL2* mRNA was significantly induced, and there were mild (but not statistically significant) increases in *CXCL1*, *CXCL10*, *CCL5*, and *IL-8* in psoriasis-associated mutant CARD14 transfected HDBECs compared to wild-type HDBECs (Fig S7 in File S1). Specifically, the E138A mutation resulted in slightly elevated expression of IL-8 and CCL5, and the G117S mutation resulted in slight upregulation of CXCL10 and CXCL1 expression.

Endothelial cells can express adhesion molecules, such as E-selectin, P-selectin, ICAM, and VCAM, which are important for infiltration of immune cells. Although the mRNA for the cell adhesion molecules was not upregulated by overactive CARD14 mutations (Figure S8a in File S1), expression could be post-transcriptionally or post-translationally regulated. Therefore, protein expression for the cell surface adhesion molecules was assessed by flow cytometry. After stimulation with TNFα, E138A transfected HDBECs had modestly increased surface expression of E-selectin, ICAM, and VCAM compared to wild-type CARD14 transfected HDBECs (Figure S8b in File S1). P-selectin was not differentially expressed between wild-type and E138A CARD14 transfected HDBECs.

### Overactive CARD14-modulated chemokines were increased in lesional skin and co-localized with endothelial cells

**Figure 5:**
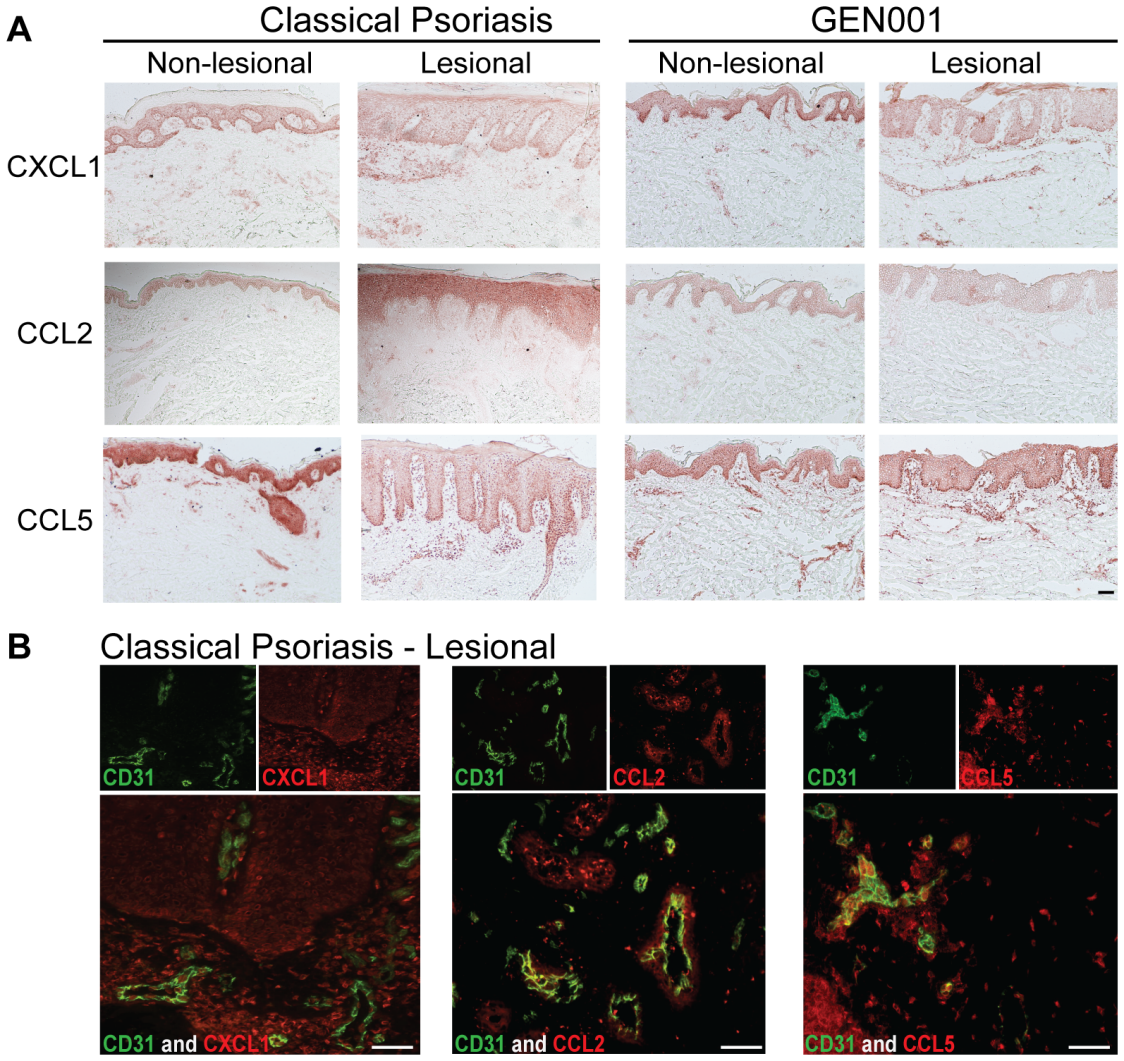
Increased expression of CARD14-modulated chemokines in psoriatic skin co-localized with dermal endothelial cells. (a) Immunohistochemistry of CXCL1, CCL2, and CCL5 in non-lesional and lesional skin of both classical psoriasis (left, representative images) and patient GEN001 (right). (b) Two-color immunofluorescence of these chemokines (red) and CD31+ endothelial cells (green) in classical psoriasis lesional skin. Representative images; bar = 10 µm.

Several of the chemokines determined to be upregulated in ECs via mutant CARD14 have been previously identified as significantly upregulated genes and proteins in psoriasis, on several platforms [16]. To determine if our *in vitro* HDBEC CARD14 transfection findings could be supported *in vivo*, skin biopsies were stained for chemokines by immunohistochemistry. Indeed, LS skin contained significantly more CCL2, CCL5, and CXCL1 compared to NL skin, in both classical psoriasis and the GEN001 patient (Figure 5a). Two-color immunofluorescence studies confirmed that dermal CXCL1, CCL2, and CCL5 co-localized with ECs (Figure 5b). Additionally, HDBECs cultured with IL-17, TNFα, and IFNγ, three cytokines heavily implicated in psoriasis pathogenesis, resulted in upregulation of IL-8, CXCL1, CXCL10, and CCL5 (Figure S9 in File S1). In conclusion, chemokines that were upregulated by transfection of psoriasis-associated *CARD14* mutations were found both endogenously in dermal EC within psoriatic lesional skin, as well as upregulated in dermal ECs exposed to key psoriatic cytokines. This suggests a connection between CARD14-NF-κB activity in endothelial cells and subsequent chemokine induction in psoriasis, and a potential role for CARD14 activity within endothelial cells in the pathogenesis of psoriasis.

## Discussion

Here we show that *CARD14*, located in *PSORS2*, is expressed in dermal ECs. Previous studies had described *CARD14* expression in keratinocytes, and transfection of psoriasis-associated *CARD14* mutations in KCs resulted in upregulated NF-κB signaling, and increased expression of IL-8 and CCL20 [2]. Similarly, CARD14^+^ ECs in psoriatic skin contained pNF-κB, demonstrating activation of ECs in psoriasis. Akin to the keratinocyte studies, ECs transfected with mutant *CARD14* upregulated chemokines which may play a role in psoriatic inflammation (IL-8, CXCL1, CXCL10, CCL2, CCL4, and CCL5). In summary, these data suggest that CARD14 over-activity within dermal ECs potentially endows them with the ability to recruit immune cells into the skin, initiating and maintaining psoriatic lesions.

Few studies have focused on the role of ECs in human psoriasis pathogenesis. Several of the chemokines that were determined to be upregulated by psoriasis-associated *CARD14* mutations in ECs (Figure 4 and Figure S7 in File S1), have been found by previous gene array studies that LS skin contains significantly higher expression of *IL-8*, *CXCL1*, and *CXCL10* than does NL skin [17]. Additionally, serum protein levels of CCL2 and CCL5 were significantly increased in psoriatic individuals compared to healthy controls [16], [17]. The chemokines found upregulated included those that are chemotactic to T-cells (CCL5, CXCL10), monocytes (CCL2, CCL4), and neutrophils (IL-8, CXCL1). Psoriatic skin is characterized by increased vascularity and angiogenesis [18], [19], alluding to the importance of blood vessels and ECs in psoriasis pathogenesis. The fact that ECs can express adhesion molecules and chemokines warrants further studies to determine their precise contribution to inflammation in psoriasis.

The roles of individual CARDs within particular cell types remains to be more specifically defined. In our studies, it was determined that both KCs and ECs express *CARD14* mRNA. However, keratinocytes expressed higher levels of CARD14 compared to endothelial cells, possibly indicating a more prominent role for CARD14 in KCs than ECs in psoriasis pathogenesis. However, *CARD10* was also detected in both KCs and ECs, and *CARD10* was expressed at a higher level than *CARD14* in both cell types. Considering the strong relationship between psoriasis and CARD14, this points to the importance of CARD14, even if expressed at lower levels. Additionally, splice variants of CARD14 have been described, such as CARD-less CARD14 (*CARMA2cl*), and a variant lacking the SH3 and GuK domains (*CARMAsh*) [7]. Both *CARMAfl* and *CARMAsh* are endogenously expressed in many cell types [7], with the *CARMAsh* variant as the most abundant in skin [2].

A recent study determined that one of the identified “psoriasis-associated” mutations in *CARD14*, was also causative for another inflammatory skin disease, familial pityriasis rubra pilaris (PRP) [10]. These mutations also led to increased activated NF-κB in the epidermis [10]. PRP is traditionally characterized as distinct from psoriasis, and therefore it is not yet understood how *CARD14* mutations can result in two disease phenotypes. Mutations in CARDs have been associated with many diseases, including cancer, autoimmunity, allergy, and susceptibility to infection [5], [20], [21]. Recently, mutations in CARD9 were found to be the genetic cause of deep dermatophytosis [22]. Therefore, NF-κB signaling through CARDs is an important topic, relevant to many human diseases.

Although we found CARD14^+^ ECs contained phosphorylated NF-κB in psoriatic skin, the important biological mediators eliciting NF-κB activation *in vivo* within dermal ECs are not yet known. Many cytokines present in the psoriatic milieu, such as IL-1 and TNF, induce NF-κB pathway activation in ECs [23]. However, it is highly possible that other molecules are eliciting CARD14-mediated NF-κB activation within ECs *in vivo*. The CARD10-Bcl10-MALT1 complex plays a crucial role in endothelial cell response to thrombin and angiotensin II via NF-κB [24], [25]. In the angiotensin II study, knockout of Bcl10 (which can also interact with CARD14 [7], [8]), resulted in reduced expression of the chemokines *CCL2* and *CXCL1* [25]. Interestingly, in our study these two chemokines were found to be upregulated by psoriasis-associated overactive CARD14 transfection in ECs. Therefore, signaling through CARDs in ECs appears to be important in inducing the expression of pro-inflammatory chemokines. The endogenous elicitors of NF-κB signaling specifically through CARD14 however, remains to be determined, and is a critical next step in understanding how CARD14 mutations contribute to psoriasis.

In conclusion, we have determined that CARD14, the gene responsible for *PSORS2*, is expressed dermally in both blood and lymphatic ECs, as well as in aortic ECs, the latter of which may prove to be an interesting link to the cardiovascular comorbidities associated with psoriasis. Transfection of psoriasis-associated CARD14 mutations into HDBECs resulted in increased protein and mRNA of several chemokines, as well as a mild increase in cell adhesions. Additionally, we were able to support our *in vitro* findings from transfected HDBECs within human skin biopsies *in vivo*. These data support the hypothesis that CARD14 is not only an important player in keratinocytes, but could also play a pro-inflammatory role within dermal ECs, a cell type which may contribute in previously unappreciated ways to psoriasis pathogenesis.

## Materials and Methods

### Skin, Aorta, Blood Samples, and Ethics Statement

Skin biopsies from normal volunteers and psoriasis patients, as well as blood samples from normal volunteers, were obtained under a Rockefeller University Institutional Review Board-approved protocol. Written informed consent was obtained and the study was performed in adherence with the Declaration of Helsinki. Samples from the GEN001 patient were collected as previously described [2], and aortic specimens were collected from cadaveric donors under a Rockefeller University Institutional Review Board-approved protocol, with the need for consent waived. Normal blood samples were used to obtain T-cells and monocytes. T-cells were isolated using RosetteSep (Stem Cell Technologies, Vancouver, British Columbia, Canada), and monocytes were isolated utilizing CD14+ magnetic selection (Militenyi Biotec, Bergisch Gladbach, Germany), both following manufactures instructions.

### Immunohistochemistry and immunofluorescence

Immunohistochemistry and immunofluorescence were performed on frozen sections of skin and aorta. Samples were fixed in acetone for 5 minutes, followed by blocking for non-specific binding with 10% serum (from the species of the secondary antibody). Sections were stained overnight at 4°C, with primary antibody diluted in 1% serum. Sections were thoroughly washed in PBS, followed by staining with secondary antibody for 30 minutes at room temperature (all secondary antibodies used at 1∶200 dilution). Sections were thoroughly washed in PBS. For immunohistochemistry, endogenous peroxide activity was quenched using 0.3% H_2_O_2_ for 20 minutes, followed by 30 minute room temperature incubation with Vectastain ABC reagent, following manufactures recommendations (Vector Laboratories, Peterborough, UK). Sections were thoroughly washed in PBS, and were developed using 3-amino-9-ethylacarbazole (AEC) diluted in acetate buffer, and activated with 30% H_2_O_2_ prior to addition to sections. Details of the antibodies and concentrations used are listed in Table S1 in File S1. For immunohistochemistry and immunofluorescence experiments, N = 3 or more per group, and representative pictures are shown. For triple-color immunofluorescence staining, single-labeled controls were used to obtain images within each laser (Figure S10 in File S1). Confocal images were acquired on a Zeiss LSM 510 confocal microscope.

### Reverse transcription-PCR

Quantitative RT-PCR was performed on samples as previously described [26]. Primers were purchased from Applied Biosystems (Carlsbad, CA); *CARD9* (Hs00364485_m1), *CARD10* (Hs00367225_m1), *CARD11* (Hs01060620_m1), *CARD14* (Hs00364499_m1), *IL-8* (Hs99999034_m1), *CCL2* (Hs00234140_m1), *CCL5* (Hs00174575_m1), *CXCL1* (Hs00236937_m1), *CXCL10* (IP10) (Hs99999049_m1), *ICAM1* (Hs00164932_m1), *VCAM* (Hs01003372_m1), *E-selectin* (Hs00950401_m1), *P-selectin* (Hs008927900). Gene expression was normalized to the house-keeping gene, human acidic ribosomal protein (hARP).

### Cell Culture and Transfection

Mutated over-active CARD14 (E138A and G117S), as well as wild-type CARD14 expression plasmids (full-length (fl) and short (sh)) (REF) were described elsewhere [2]. HDBECs (PromoCell, Heidelberg, Germany) were cultured as previously described [27]. HDBECs were transfected using PromoFectin-HUVEC according to manufactures recommendations. In brief, HDBECs were seeded into a 24 well plate and allowed to reach 50%-70% confluency. PromoFectin-HUVEC and expression plasmid DNA were diluted in serum-free Endothelial Cell Basal Medium (ECBM) (PromoCell). 2 ul of PromoFectin-HUVEC was used per 1 ug of plasmid DNA, and allowed to incubate at room temperature for 30 minutes. HDBECs were washed with ECBM. Transfections took place in 500 ul of serum-free medium for 6 hours, after which media was replaced with EC media containing serum. Using this method, transfection efficiency of HDBEC was 60–65%, as determined by a GFP-expression plasmid and subsequent flow cytometry analysis. For stimulation with TNFα (25 ng/mL), cells were allowed to rest for 3 hours after transfection. RNA and supernatant were collected 24 hours post-transfection.

### Flow Cytometry

Transfected HDBECs were stimulated overnight with TNFα (25 ng/mL) and collected using a 5 mM EDTA-HBSS plus 10 mM HEPES solution for 2 minutes at 37°C. Cells were resuspended 2%FBS-PBS, and stained with Aquamarina LIVE-DEAD stain, and the following antibodies: VCAM-1 (BD Pharmingen, Clone 51-10C9), P-selectin (BD Pharmingen, Clone AK-4), ICAM (Thermo Scientific, Clone 1H4), and E-selectin (BD Pharmingen, Clone 68-5H11). Data was analyzed using FlowJo software.

### Chemokine Multiplex Plate

Supernatants were assessed for chemokines using the Meso Scale (Gaithersburg, MD) Human Chemokine 7-Plex Assay Ultra Sensitive Kit according to manufactures instructions.

### Bioinformatics

The microarray data analyzed in this manuscript has been previously described and published elsewhere [2], [15], [16]. Expression data was adjusted for batch effect using ComBat [28]. Gene sets have been curated by our lab and have been used extensively [15], [16], [29]-[34]. For each gene set, a score was obtained using the gene set variation analysis (GSVA) methodology [35].

### Statistics

Quantitative RT-PCR and Multiplex Chemokine Assay results from transfection experiments were analyzed using an unpaired, 2-tailed t-test. P-values<0.05 were considered statistically significant. *CARD14fl* and *CARD14sh* transfected HDBECs behaved highly similarly (p-values ranged from 0.25-0.76), therefore these groups were combined to create the WT CARD14 group.

## Supporting Information

File S1 **Supporting files.** Figure S1–Figure S10. Table S1.
(PDF)

## References

[1] Lowes MA, Bowcock AM, Krueger JG (2007) Pathogenesis and therapy of psoriasis. Nature 445: 866–873. 17314973 10.1038/nature05663

[2] Jordan CT, Cao L, Roberson ED, Pierson KC, Yang CF, et al (2012) PSORS2 is due to mutations in CARD14. Am J Hum Genet 90: 784–795. 22521418 10.1016/j.ajhg.2012.03.012 PMC3376640

[3] Tsoi LC, Spain SL, Knight J, Ellinghaus E, Stuart PE, et al (2012) Identification of 15 new psoriasis susceptibility loci highlights the role of innate immunity. Nat Genet 44: 1341–1348. 23143594 10.1038/ng.2467 PMC3510312

[4] Jabbari A, Suarez-Farinas M, Dewell S, Krueger JG (2012) Transcriptional profiling of psoriasis using RNA-seq reveals previously unidentified differentially expressed genes. J Invest Dermatol 132: 246–249. 21850022 10.1038/jid.2011.267 PMC3381505

[5] Blonska M, Lin X (2011) NF-kappaB signaling pathways regulated by CARMA family of scaffold proteins. Cell Res 21: 55–70. 21187856 10.1038/cr.2010.182 PMC3193407

[6] Hoesel B, Schmid JA (2013) The complexity of NF-kappaB signaling in inflammation and cancer. Mol Cancer 12: 86. 23915189 10.1186/1476-4598-12-86 PMC3750319

[7] Scudiero I, Zotti T, Ferravante A, Vessichelli M, Vito P, et al (2011) Alternative splicing of CARMA2/CARD14 transcripts generates protein variants with differential effect on NF-kappaB activation and endoplasmic reticulum stress-induced cell death. J Cell Physiol 226: 3121–3131. 21302310 10.1002/jcp.22667 PMC3229840

[8] Bertin J, Wang L, Guo Y, Jacobson MD, Poyet JL, et al (2001) CARD11 and CARD14 are novel caspase recruitment domain (CARD)/membrane-associated guanylate kinase (MAGUK) family members that interact with BCL10 and activate NF-kappa B. J Biol Chem. 276: 11877–11882. 10.1074/jbc.M010512200 11278692

[9] Jordan CT, Cao L, Roberson ED, Duan S, Helms CA, et al (2012) Rare and common variants in CARD14, encoding an epidermal regulator of NF-kappaB, in psoriasis. Am J Hum Genet 90: 796–808. 22521419 10.1016/j.ajhg.2012.03.013 PMC3376540

[10] Fuchs-Telem D, Sarig O, van Steensel MA, Isakov O, Israeli S, et al (2012) Familial pityriasis rubra pilaris is caused by mutations in CARD14. Am J Hum Genet 91: 163–170. 22703878 10.1016/j.ajhg.2012.05.010 PMC3397268

[11] Boehncke WH, Boehncke S, Tobin AM, Kirby B (2011) The 'psoriatic march': a concept of how severe psoriasis may drive cardiovascular comorbidity. Exp Dermatol 20: 303–307. 21410760 10.1111/j.1600-0625.2011.01261.x

[12] Madonna S, Scarponi C, Pallotta S, Cavani A, Albanesi C (2012) Anti-apoptotic effects of suppressor of cytokine signaling 3 and 1 in psoriasis. Cell Death Dis 3: e334. 22739986 10.1038/cddis.2012.69 PMC3388239

[13] Lizzul PF, Aphale A, Malaviya R, Sun Y, Masud S, et al (2005) Differential expression of phosphorylated NF-kappaB/RelA in normal and psoriatic epidermis and downregulation of NF-kappaB in response to treatment with etanercept. J Invest Dermatol 124: 1275–1283. 15955104 10.1111/j.0022-202X.2005.23735.x

[14] Suarez-Farinas M, Lowes MA, Zaba LC, Krueger JG (2010) Evaluation of the psoriasis transcriptome across different studies by gene set enrichment analysis (GSEA). PLoS One 5: e10247. 20422035 10.1371/journal.pone.0010247 PMC2857878

[15] Tian S, Krueger JG, Li K, Jabbari A, Brodmerkel C, et al (2012) Meta-analysis derived (MAD) transcriptome of psoriasis defines the "core" pathogenesis of disease. PLoS One 7: e44274. 22957057 10.1371/journal.pone.0044274 PMC3434204

[16] Lowes MA, Suárez-Fariñas M, Krueger JG (2014) Immunology of Psoriasis. Annual Review in Immunology 32.. 10.1146/annurev-immunol-032713-120225 PMC4229247 24655295

[17] Suarez-Farinas M, Li K, Fuentes-Duculan J, Hayden K, Brodmerkel C, et al (2012) Expanding the psoriasis disease profile: interrogation of the skin and serum of patients with moderate-to-severe psoriasis. J Invest Dermatol 132: 2552–2564. 22763790 10.1038/jid.2012.184 PMC3472561

[18] Heidenreich R, Rocken M, Ghoreschi K (2009) Angiogenesis drives psoriasis pathogenesis. Int J Exp Pathol 90: 232–248. 19563608 10.1111/j.1365-2613.2009.00669.x PMC2697548

[19] Canavese M, Altruda F, Ruzicka T, Schauber J (2010) Vascular endothelial growth factor (VEGF) in the pathogenesis of psoriasis—a possible target for novel therapies? J Dermatol Sci 58: 171–176. 20430590 10.1016/j.jdermsci.2010.03.023

[20] Roth S, Ruland J (2013) Caspase recruitment domain-containing protein 9 signaling in innate immunity and inflammation. Trends Immunol 34: 243–250. 23523010 10.1016/j.it.2013.02.006

[21] Nakamura S, Nakamura S, Matsumoto T, Yada S, Hirahashi M, et al (2005) Overexpression of caspase recruitment domain (CARD) membrane-associated guanylate kinase 1 (CARMA1) and CARD9 in primary gastric B-cell lymphoma. Cancer 104: 1885–1893. 16177990 10.1002/cncr.21421

[22] Lanternier F, Pathan S, Vincent QB, Liu L, Cypowyj S, et al (2013) Deep dermatophytosis and inherited CARD9 deficiency. N Engl J Med 369: 1704–1714. 24131138 10.1056/NEJMoa1208487 PMC4084693

[23] Pober JS, Sessa WC (2007) Evolving functions of endothelial cells in inflammation. Nat Rev Immunol 7: 803–815. 17893694 10.1038/nri2171

[24] Delekta PC, Apel IJ, Gu S, Siu K, Hattori Y, et al (2010) Thrombin-dependent NF-{kappa}B activation and monocyte/endothelial adhesion are mediated by the CARMA3.Bcl10.MALT1 signalosome. J Biol Chem 285: 41432–41442. 21041303 10.1074/jbc.M110.158949 PMC3009869

[25] McAllister-Lucas LM, Jin X, Gu S, Siu K, McDonnell S, et al (2010) The CARMA3-Bcl10-MALT1 signalosome promotes angiotensin II-dependent vascular inflammation and atherogenesis. J Biol Chem 285: 25880–25884. 20605784 10.1074/jbc.C110.109421 PMC2923974

[26] Johnson-Huang LM, Suarez-Farinas M, Sullivan-Whalen M, Gilleaudeau P, Krueger JG, et al (2010) Effective narrow-band UVB radiation therapy suppresses the IL-23/IL-17 axis in normalized psoriasis plaques. J Invest Dermatol 130: 2654–2663. 20555351 10.1038/jid.2010.166 PMC2955161

[27] Belkin DA, Mitsui H, Wang CQ, Gonzalez J, Zhang S, et al (2013) CD200 upregulation in vascular endothelium surrounding cutaneous squamous cell carcinoma. JAMA Dermatol 149: 178–186. 23560298 10.1001/jamadermatol.2013.1609

[28] Johnson WE, Li C, Rabinovic A (2007) Adjusting batch effects in microarray expression data using empirical Bayes methods. Biostatistics 8: 118–127. 16632515 10.1093/biostatistics/kxj037

[29] Nograles KE, Zaba LC, Guttman-Yassky E, Fuentes-Duculan J, Suarez-Farinas M, et al (2008) Th17 cytokines interleukin (IL)-17 and IL-22 modulate distinct inflammatory and keratinocyte-response pathways. Br J Dermatol 159: 1092–1102. 18684158 10.1111/j.1365-2133.2008.08769.x PMC2724264

[30] Krueger JG, Fretzin S, Suarez-Farinas M, Haslett PA, Phipps KM, et al. (2012) IL-17A is essential for cell activation and inflammatory gene circuits in subjects with psoriasis. J Allergy Clin Immunol 130: : 145–154 e149. 10.1016/j.jaci.2012.04.024 PMC3470466 22677045

[31] Mee JB, Johnson CM, Morar N, Burslem F, Groves RW (2007) The psoriatic transcriptome closely resembles that induced by interleukin-1 in cultured keratinocytes: dominance of innate immune responses in psoriasis. Am J Pathol 171: 32–42. 17591951 10.2353/ajpath.2007.061067 PMC1941577

[32] Chiricozzi A, Guttman-Yassky E, Suarez-Farinas M, Nograles KE, Tian S, et al (2011) Integrative responses to IL-17 and TNF-alpha in human keratinocytes account for key inflammatory pathogenic circuits in psoriasis. J Invest Dermatol 131: 677–687. 21085185 10.1038/jid.2010.340

[33] Suarez-Farinas M, Arbeit R, Jiang W, Ortenzio FS, Sullivan T, et al (2013) Suppression of Molecular Inflammatory Pathways by Toll-Like Receptor 7, 8, and 9 Antagonists in a Model of IL-23-Induced Skin Inflammation. PLoS One 8: e84634. 24386404 10.1371/journal.pone.0084634 PMC3874038

[34] Chiricozzi A, Nograles KE, Johnson-Huang LM, Fuentes-Duculan J, Cardinale I, et al (2014) IL-17 Induces an Expanded Range of Downstream Genes in Reconstituted Human Epidermis Model. PLoS One 9: e90284. 24587313 10.1371/journal.pone.0090284 PMC3938679

[35] Hanzelmann S, Castelo R, Guinney J (2013) GSVA: gene set variation analysis for microarray and RNA-seq data. BMC Bioinformatics 14: 7. 23323831 10.1186/1471-2105-14-7 PMC3618321

