# Supplementary material for: CARD14 Expression in Dermal Endothelial Cells in Psoriasis
Source: PLoS One. 2014 Nov 4;9(11):e111255. doi: 10.1371/journal.pone.0111255 (PMC4219711; doi:10.1371/journal.pone.0111255)
Supplement: File S1 — Supporting files. Figure S1–Figure S10. Table S1. (PDF) [file pone.0111255.s001.pdf]

Figure S1

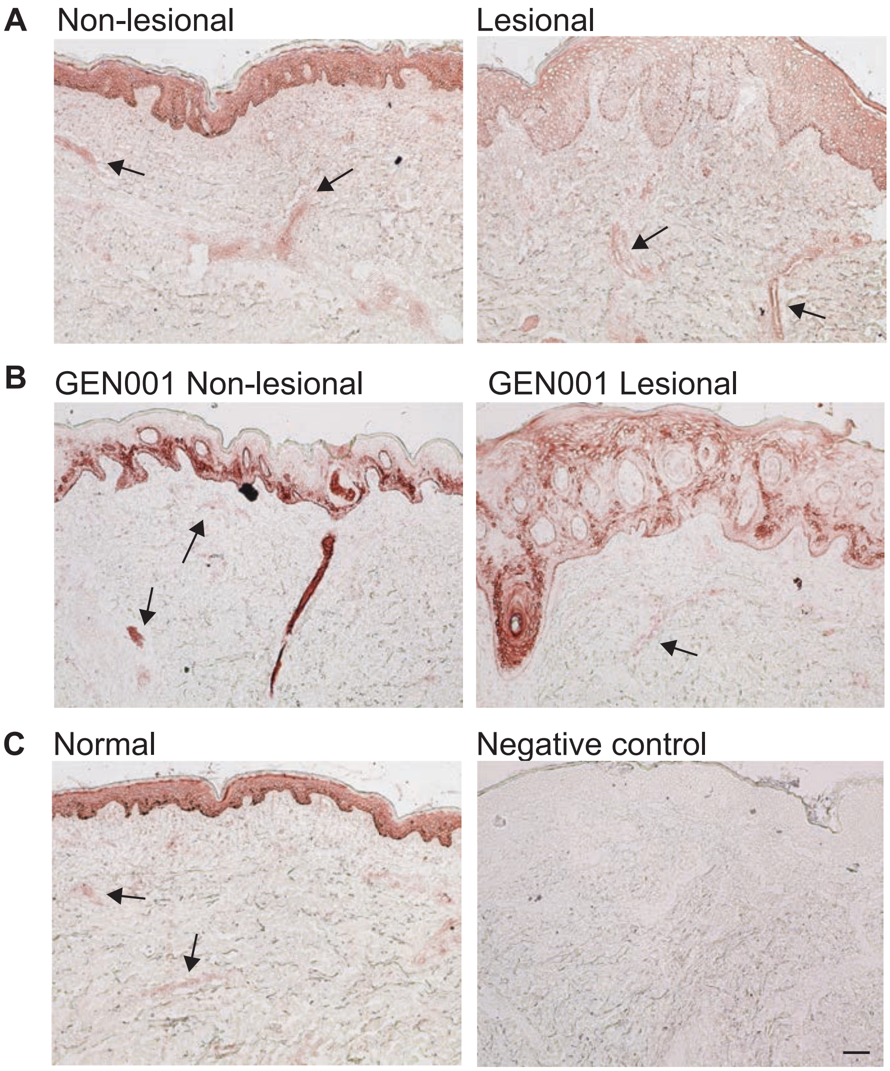

Figure S1. Dermal Populations of CARD14⁺ cells are found in human skin. Immunohistochemistry for CARD14 on fixed, frozen skin sections of (A) non-lesional and lesional classical psoriasis, (B) non-lesional and lesional skin from patient GEN001, with a confirmed over-active mutation in CARD14 (Jordan CT et al, Am J Hum Genet, 2012 May 4;90(5):784; PMID22521418), and (C) normal human skin. Lower right image is the negative staining control, performed in classical psoriasis lesional skin. Arrows point to dermal CARD14⁺ cells. Representative images are shown, bar = 10μm.

Figure S2

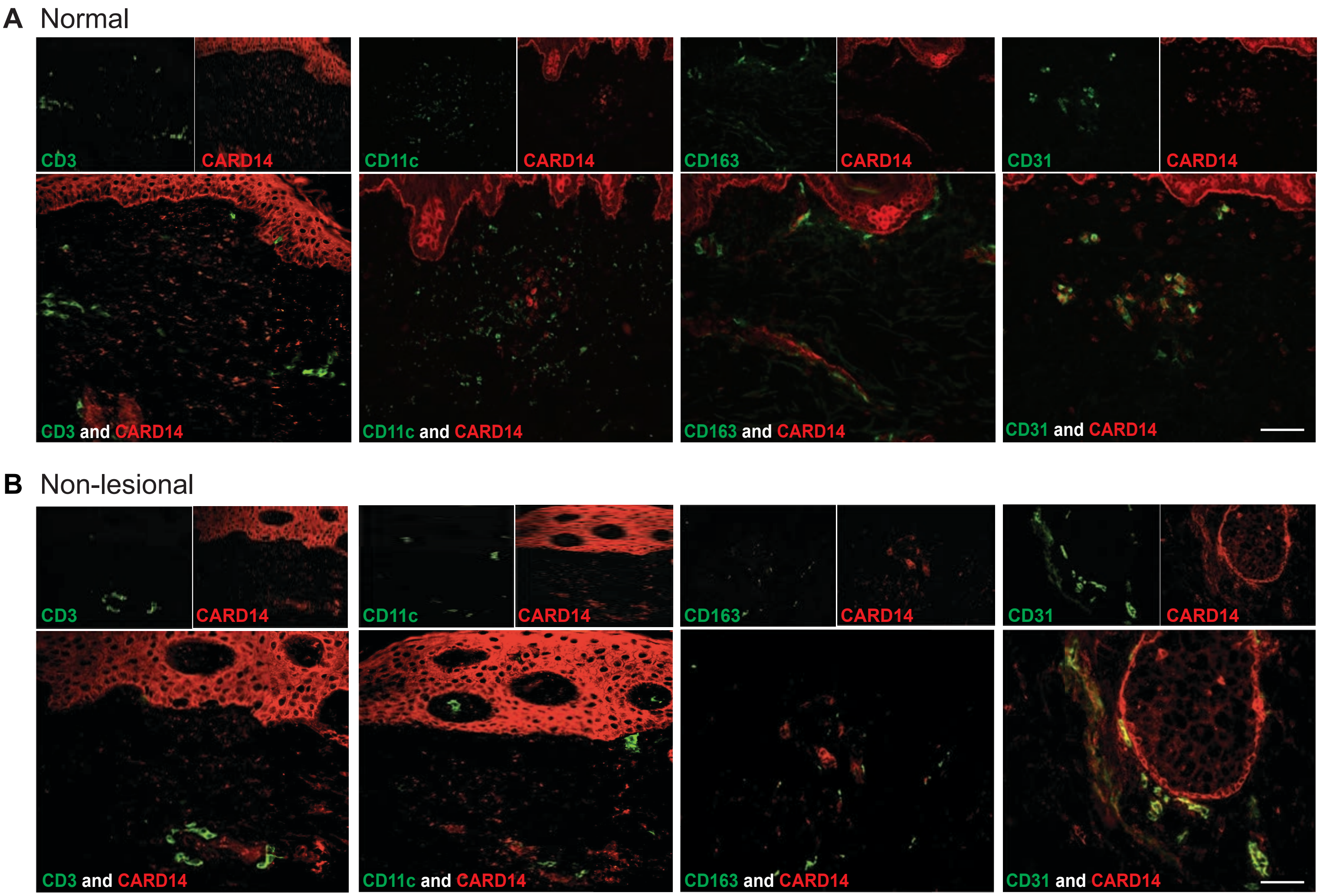

Figure S2. Dermal CARD14 is expressed in endothelial cells in normal and non-lesional skin. Two color immunofluroscence on frozen skin sections of (A) normal and (B) non-lesional skin demonstrates that CARD14 (red) does not colocalize with various cell markers (green); CD3 (T-cells), CD11c (dendritic cells), and CD163 (macrophages), but does colocalize with $CD31^+$ endothelial cells (green) in both normal and non-lesional skin. Representative images shown; bar = 10μm.

Figure S3

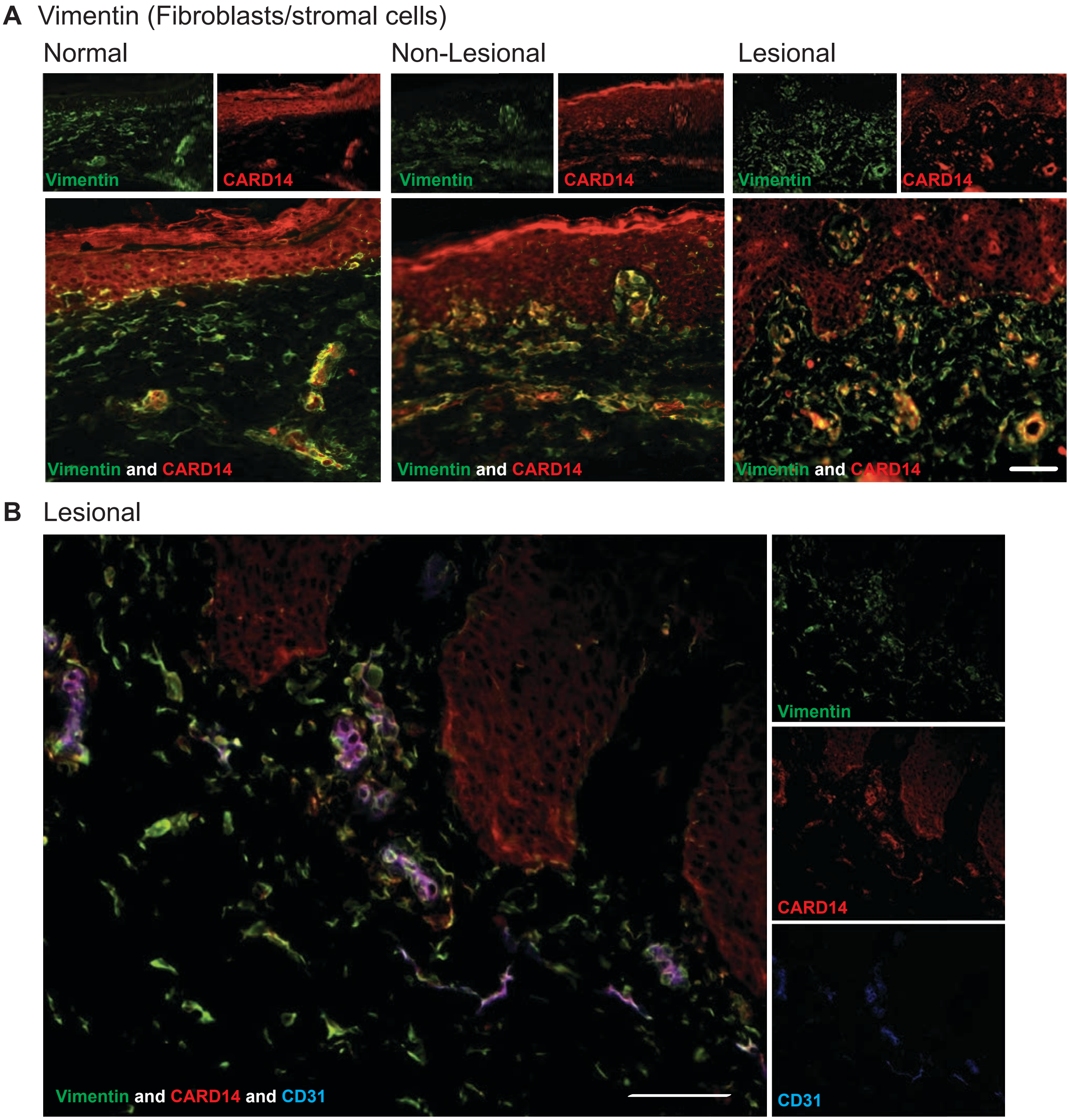

Figure S3. Dermal CARD14 colocalizes with vimentin^{+}CD31^{+} cells. (A) Two-color immunofluroscence demonstrates CARD14 (red) colocalizes with some vimentin (green) cells in normal, non-lesional, and lesional skin. (B) Triple-color immunofluroscence staining in lesional skin demonstrates that the dermal CARD14^{+} (red) and vimentin^{+}(green), colocalize with the CD31^{+} endothelial cells (blue). Representative image shown; bar = 10μm.

Figure S4

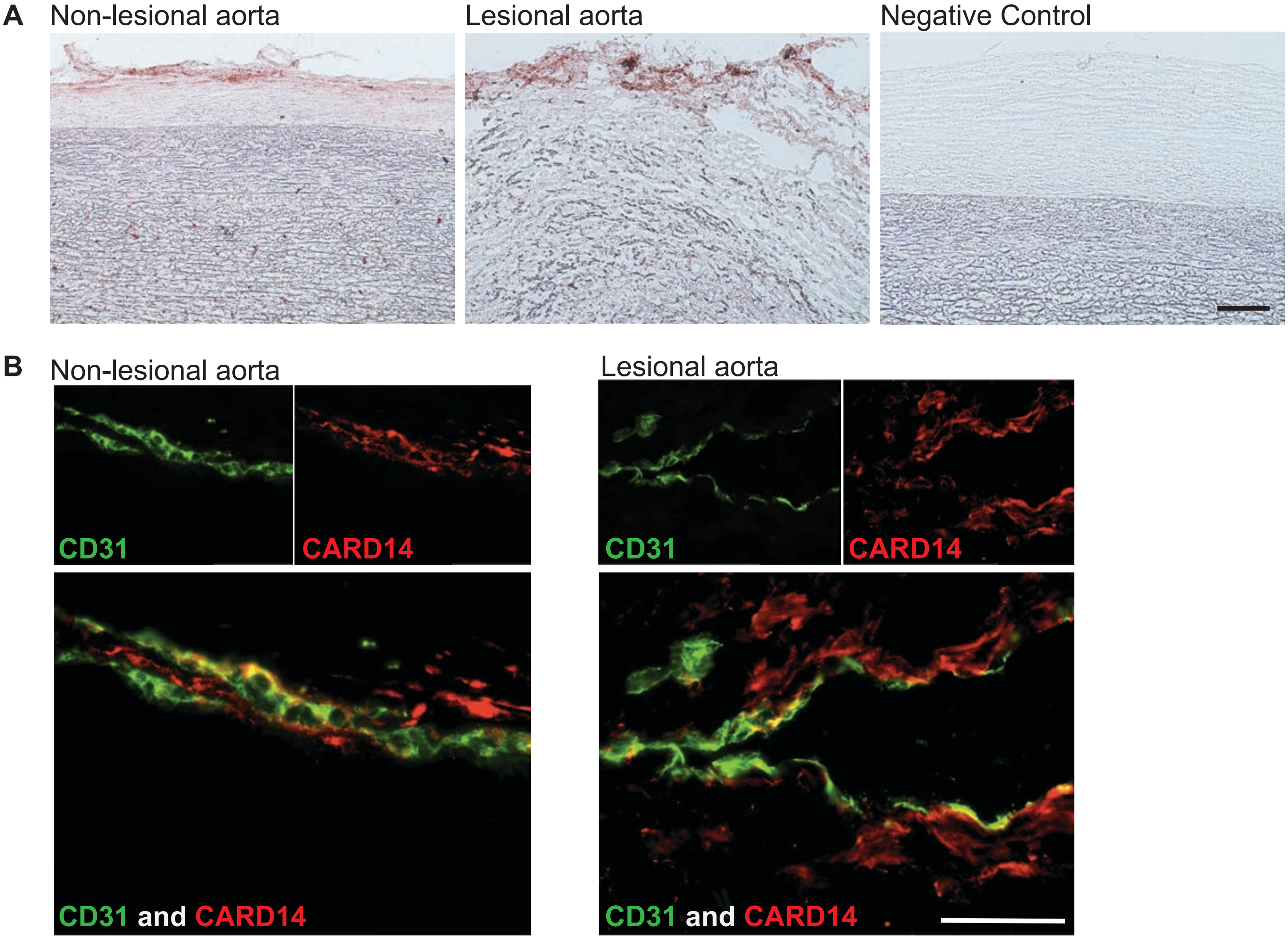

Figure S4. CARD14 is also expressed in aortic endothelial cells. (A) Immunohistochemistry for CARD14 on fixed, frozen sections of aorta, obtained from cadavers with atheleroscletotic disease, classified as non-lesional (normal apearing) or lesional (atherlosclerotic) based on macroscopic observation. Negative control shown far right. (B) Two-color immunofluroscence for CD31 (green) and CARD14 (red) in aorta. Representative images are shown; bar = 10μm.

Figure S5

A

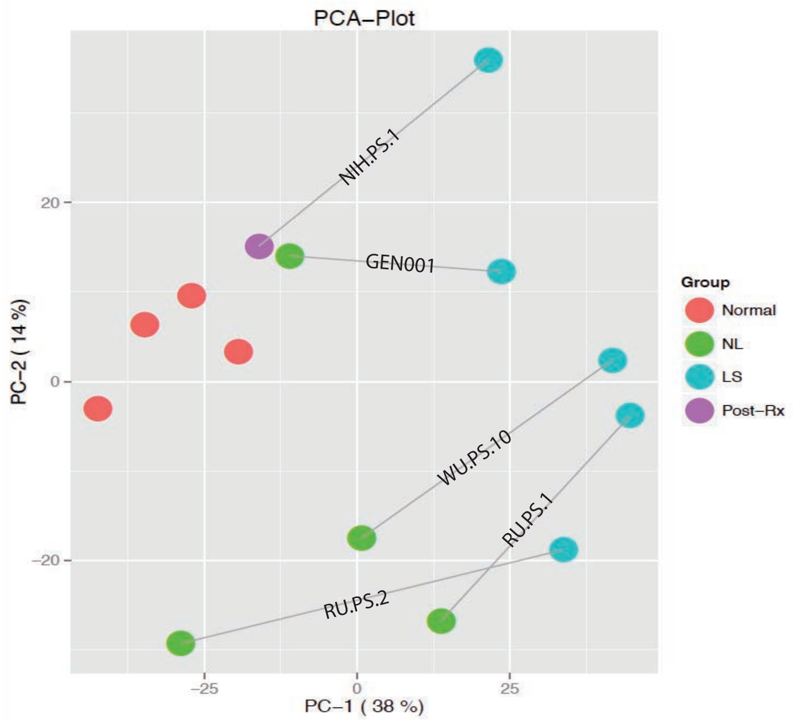

B

Published Psoriasis Gene Sets Enriched in GEN001 (*CARD14* mutation) LS vs NL Transcriptome

Name	Size	ES	NES	NOM p-val	FDR q-val	CS
Psoriasis_Gudjonsson UP	494	0.82	4.17	0	0	0.78
Psoriasis_Gudjonsson DOWN	242	-0.74	-3.55	0	0	
Psoriasis_Yao UP	952	0.74	3.95	0	0	0.71
Psoriasis_Yao DOWN	829	-0.68	-3.63	0	0	
Psoriasis_SF UP	568	0.77	3.94	0	0	0.75
Psoriasis_SF DOWN	686	-0.73	-3.83	0	0	
Psoriasis_Bowcock UP	261	0.77	3.69	0	0	0.70
Psoriasis_Bowcock DOWN	388	-0.63	-3.13	0	0	

Published Psoriasis Gene Sets Enriched in NIH(*CARD14* mutation) LS vs post-Rx Transcriptome

Name	Size	ES	NES	NOM p-val	FDR q-val	CS
Psoriasis_Gudjonsson UP	494	0.82	3.90	0	0	0.78
Psoriasis_Gudjonsson DOWN	242	-0.74	-3.43	0	0	
Psoriasis_Yao UP	568	0.78	3.81	0	0	0.71
Psoriasis_Yao DOWN	686	-0.64	-3.19	0	0	
Psoriasis_SF UP	952	0.74	3.75	0	0	0.69
Psoriasis_SF DOWN	829	-0.63	-3.21	0	0	
Psoriasis_Bowcock UP	261	0.78	3.51	0	0	0.65
Psoriasis_Bowcock DOWN	388	-0.52	-2.48	0	0	

Figure S5. Transcriptomic profile of patients with CARD14 muations. (A) Principle Component Analysis (PCA) for the gene expression of normal, LS, and NL or post-treatment biopsies of patients with confirmed CARD14 mutations (GEN001 and NIH.PS.1) and classical psoriasis (RU and WU samples). (B) Gene Set Enrichment Analysis (GSEA) of published psoriasis upregulated and downregulated transcriptomes (DEGs) compared to gene expression profile of patients with confirmed CARD14 mutations. cs = connectivity score. Additional information on bioinfomatics is located in the Material and Methods

Figure S6.

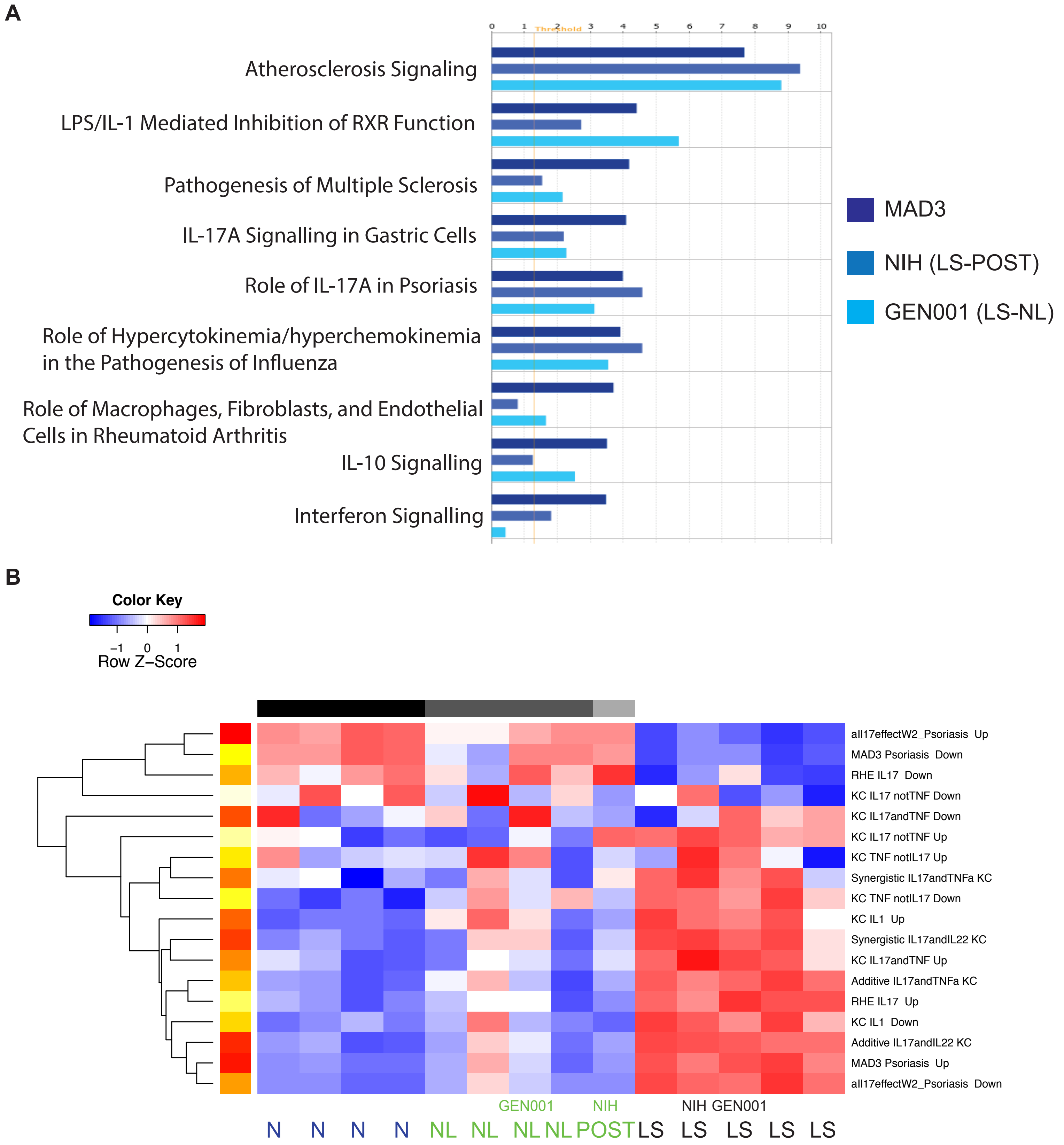

Figure S6. Transcriptomic profile of patients with CARD14 mutations. (A) Ingenuity Pathway Analysis (IPA), showing biological pathways significantly upregulated in both classical psoriasis (MAD3) as well as patients with confirmed CARD14 mutations. (B) Heat map of cyotkine GSVA (gene set variation analysis)-derived scores in normal, LS, and NL or post-treatment biopsies of patients with confirmed CARD14 mutations (GEN001 and NIH.PS.1) and classical psoriasis. Additional information on bioinformatics is located in the Materialsand Methods.

Figure S7

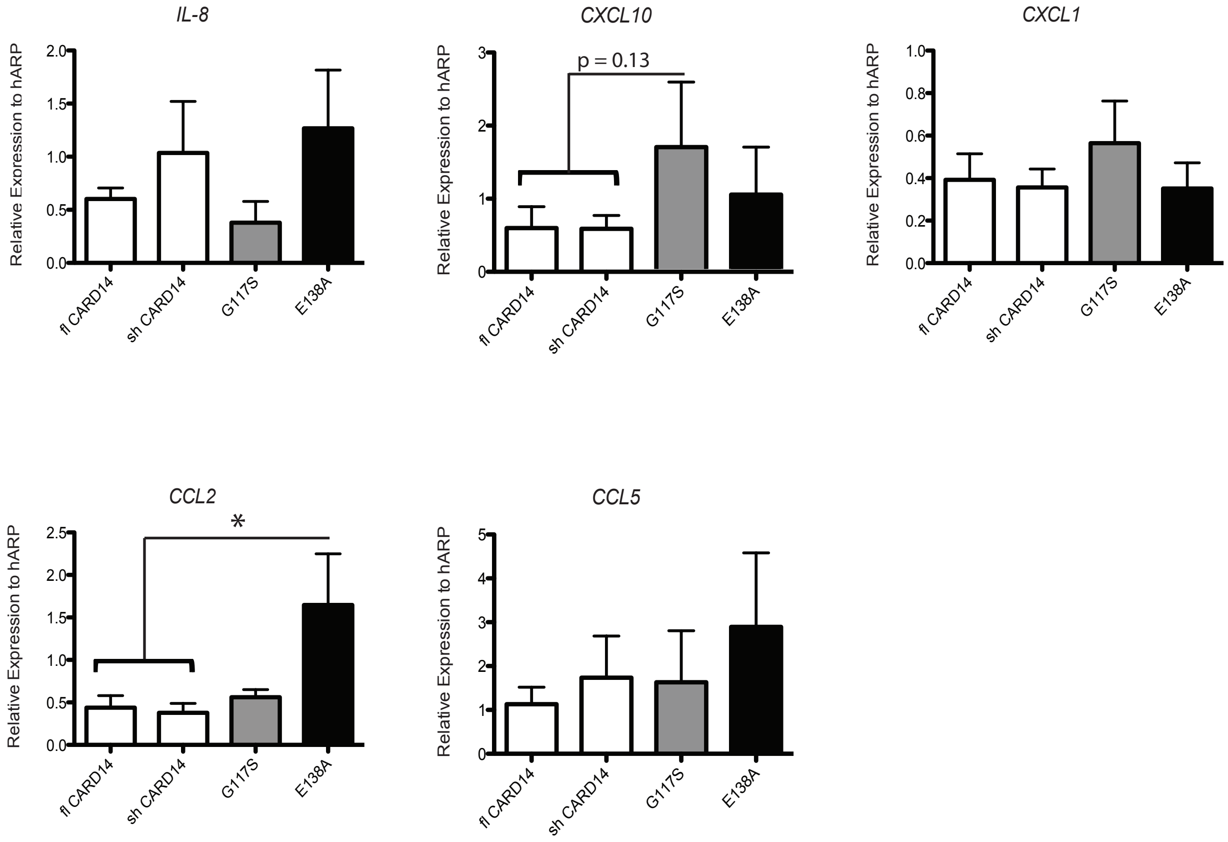

Figure S7. Transfection of psoriasis-associated CARD14 mutations into dermal endothelial cells resulted in increased expression of several chemokines. Quantitative RT-PCR for various chemokines in endothelial cells transfected with wild-type (full-length (fl) and short (sh) CARD14)(white), or psoriasis-associated CARD14 mutation constructs: G117S (gray) and E138A (black). N= 3 per group. Error bars represent the standard error of the mean.

Figure S8

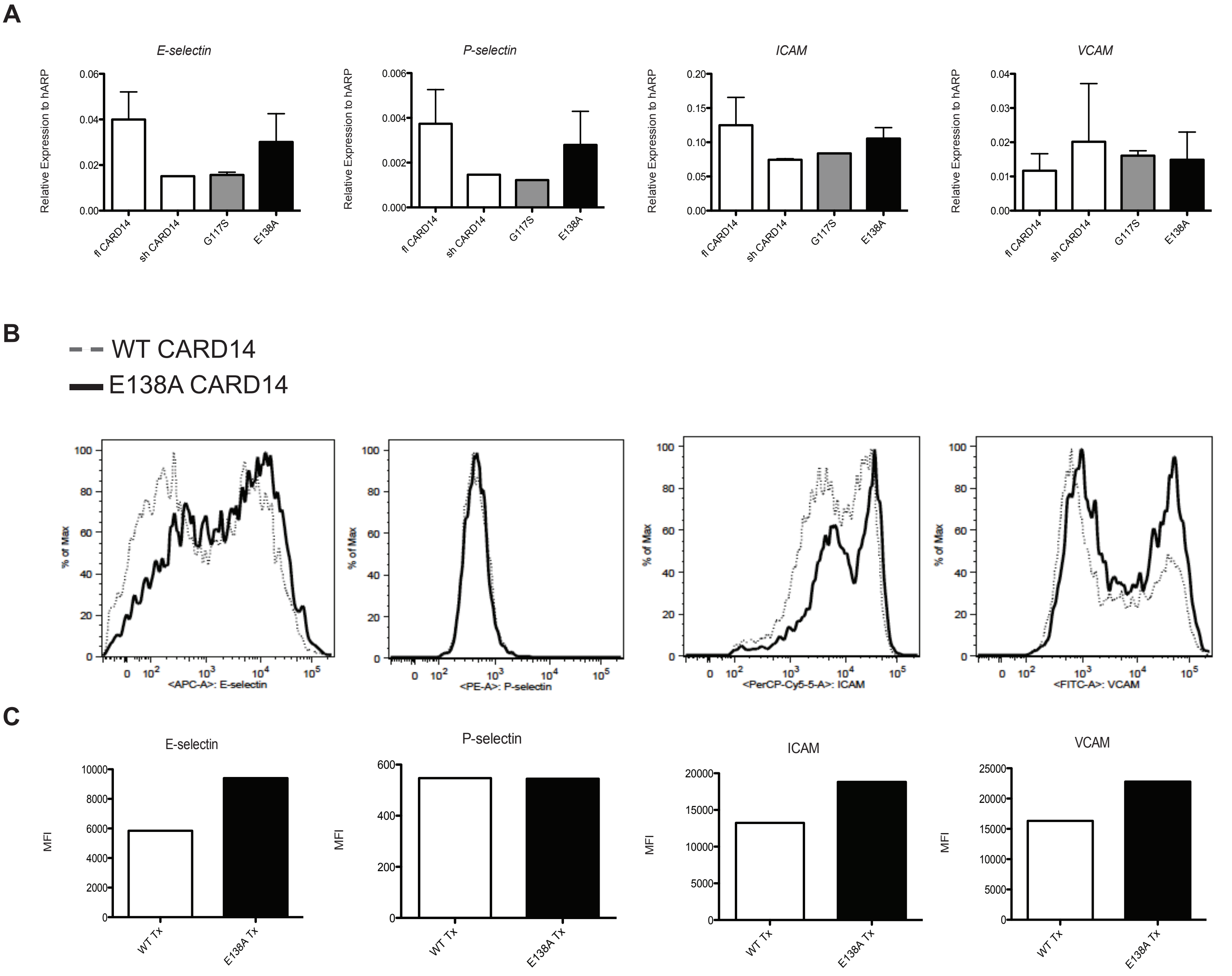

Figure S8. Minimal upregulation of cell adhesion molecule protein expression on E138A transfected HDBECs. (A) qRT-PCR analysis of E-selectin, P-selectin, ICAM, and VCAM on wild-type (fl and shCARD14) transfected HDBECs, or HDBECs transfected with psoriasis associated mutations (G117S and E138A). Expression is presented as relative to the housekeeping gene, hARP. N = 2-3 per group. Error bars represent the standard error of the mean. (B) and (C) Expanded HDBECs transfected with the wild-typeCARD14 or the E138A psoriasis-associated CARD14 mutation (N=1) were stimulated with TNF-alpha overnight and then assessed for cell surface protein expression of adhesion molecules by flow cytometry; (B) histograms and (C) corresponding mean fluroscence intensity (MFI) are shown.

Figure S9

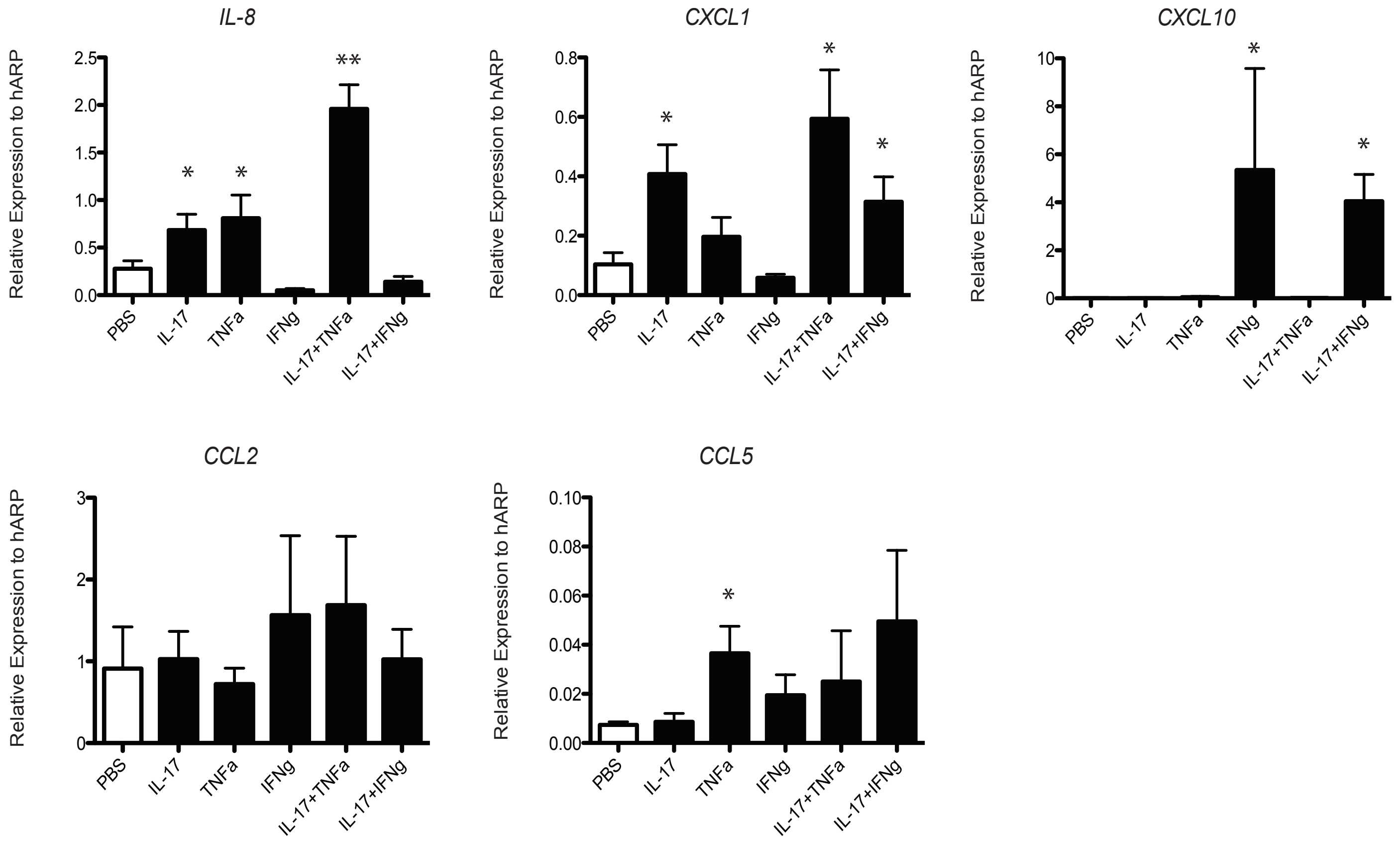

Figure S9. Psoriatic cytokines upregulate chemokines in dermal blood endothelial cells. Human dermal blood endothelial cells were cultured with either vehicle control (PBS), IL-17 (100ng/ml), TNF-alpha (20ng/ml), IFN-gamma (20ng/ml), or combinatations. RNA was isolated after 12 hours of exposure to cytokines, and expression of various chemokines was determined using qRT-PCR (as described in the materials and methods). Error bars represent the standard error of the mean. Significance was determined by comparison of cytokine treatment versus PBS, * $p < 0.05$, **$p < 0.01$ using a paired t-test. N=3.

Figure S10

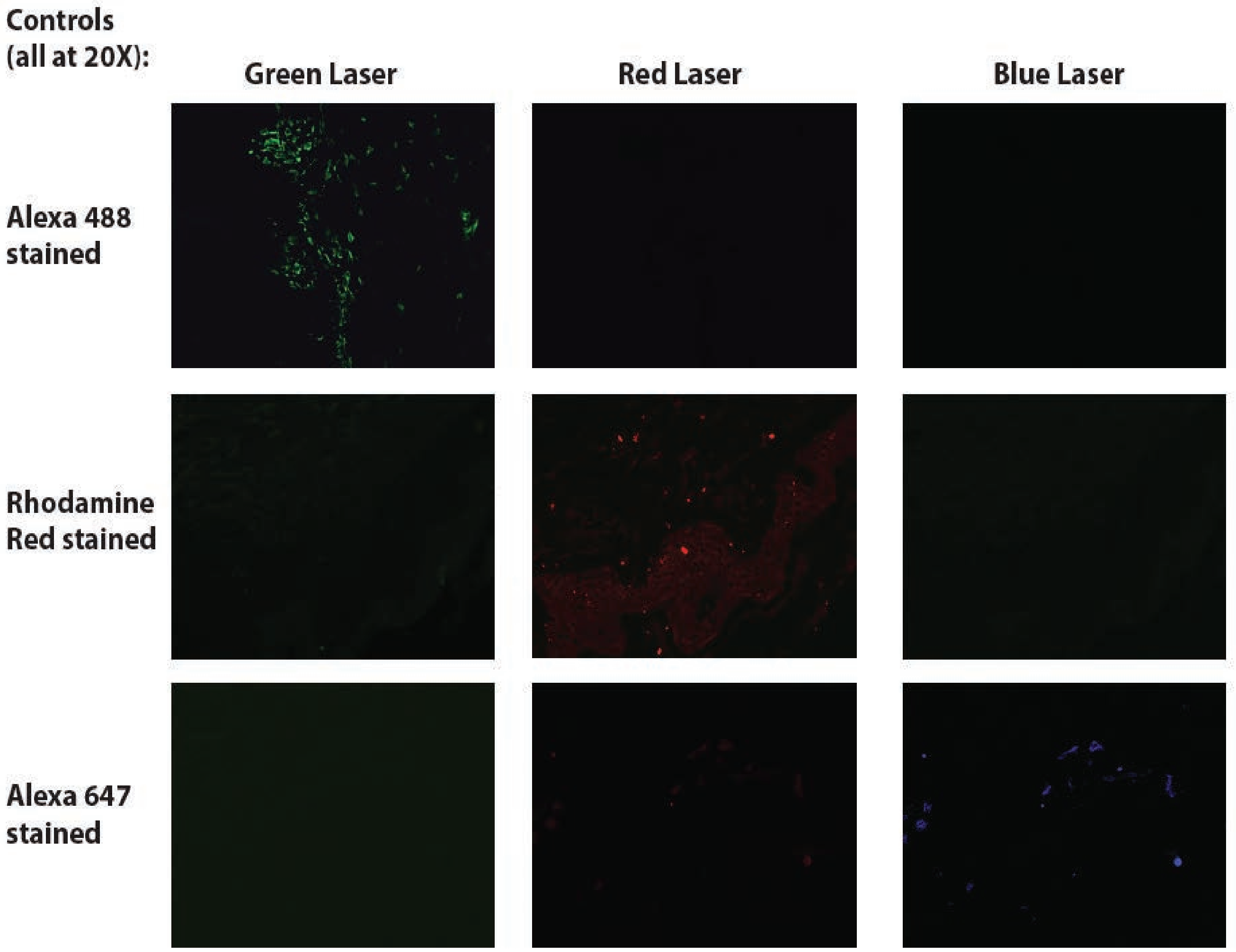

Figure S10. Single stained flurochrome slide controls for triple-color immunofluroscence studies. Representative images of each single-flurochrome stained sample using the the green, red, or blue laser are shown at 20X.

Table S1: Antibodies used for immunohistochemistry/immunofluorescence

Antigen	Manufacturer	Clone[a]	Dilution	Amplification/detection (secondary antibodies)
CARD14	Sigma-Aldrish	Rabbit polyclonal	1:600	Goat anti-rabbit Ig rhodamine red
CD3	BD	SK7	1:100	Goat anti-mouse IgG1 A-488
CD11c	BD	B-ly6	1:100	Goat anti-mouse IgG1 A-488
CD163-FITC	Acris	5C6-FAT	1:100	Goat anti-mouse IgG FITC
Vimentin	Thermo Scientific	Ab2	1:100	Goat anti-mouse IgG1 A-488
CD31	BD	WM59	1:100	Goat anti-mouse IgG1 A-488 or A-647
PAL-E	abcam	ab8853	1:50	Goat anti-mouse IgG1 A-488
LYVE-1	R&D	537028	1:200	Goat anti-mouse IgG1 A-488
pNFkB (ser276)	Santa Cruz	Rabbit polyclonal	1:100	Zenon conjugated A-647
CXCL1	Acris	Rabbit polyclonal	1:100	Goat anti-rabbit Ig rhodamine red
CCL2	Novus Biologicals	MNA1	1:100	Zenon conjugated A-568
CCL5	abcam	VL-1	1:200	Goat anti-mouse IgG2b A-568

[a] All antibodies are murine monoclonal unless otherwise stated.
